# Representations of facial expressions since Darwin

**DOI:** 10.1017/ehs.2022.10

**Published:** 2022-04-28

**Authors:** David Perrett

**Affiliations:** School of Psychology and Neuroscience, University of St Andrews, St Mary's Quad, St Andrews, Fife KY169JP, UK

**Keywords:** Expression, face, representation

## Abstract

Darwin's book on expressions of emotion was one of the first publications to include photographs (Darwin, *The expression of the emotions in Man and animals*, 1872). The inclusion of expression photographs meant that readers could form their own opinions and could, like Darwin, survey others for their interpretations. As such, the images provided an evidence base and an ‘open source’. Since Darwin, increases in the representativeness and realism of emotional expressions have come from the use of composite images, colour, multiple views and dynamic displays. Research on understanding emotional expressions has been aided by the use of computer graphics to interpolate parametrically between different expressions and to extrapolate exaggerations. This review tracks the developments in how emotions are illustrated and studied and considers where to go next.

## Graphical abstract

**Figure:**
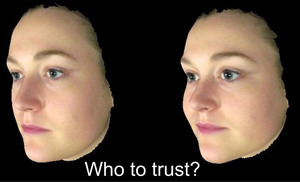

**Social media summary:** This review tracks the development in illustration and study of facial expressions of emotions.

## Introduction

Darwin wrote his book, *The expression of the emotions in Man and animals* in just four months after completing the page proofs for the *Descent of Man* (Ekman, 2009). One feature that was unusual for that time was the inclusion of photographs of facial expressions in the book. This was pioneering in the publishing world, partly because printing photographs was costly. The purpose of this review is to discuss the impact of the inclusion of photographs in Darwin's initial work and to chart the development of emotion illustrations (particularly those of facial expressions) since that time. The review will cover the utility of depictions within the discipline of psychology.

## Darwin as a psychologist

Darwin collected reactions to photographs in a way that was a prototype for many psychological experiments. He presented an image, for example, a man's face stimulated by Duchenne's electrical probes, to 20 educated persons of various ages and both sexes, and asked them what emotion or feeling the man was displaying. He recorded the participants’ answers in the words that they had used (Darwin, 1872; see Wilson, 2006: 1267). He used these psychological experiments in the narrative of *The expression of the emotions*, referring to photographic illustrations both posed and spontaneous and discussed his survey of responses to them. He concluded that the public were consistent in their interpretations: for example, 14 out of 15 people recognised a furrowed forehead as ‘despairing sorrow’, ‘suffering endurance’ or ‘melancholy’. The inclusion of photographs in his book thus invited readers to make judgments for themselves and, in this way, Darwin drew his readers into a conversation. The photographs thus represented a move towards ‘open science’ (Foster & Deardorff, 2017) because others could use the depicted expressions to verify or falsify Darwin's conclusions about the public's reactions.

**Figure 1.**
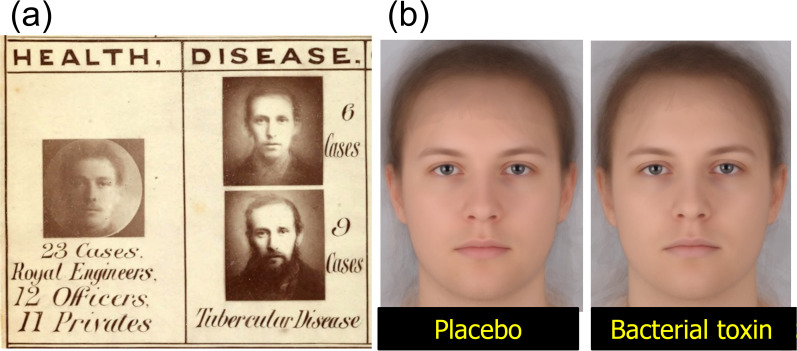
Images of health and sickness from the nineteenth and twenty-first centuries. (a) Galton's composite photographs of ‘health’ – a combination of 23 Royal Engineers, and ‘sickness’ – combinations of six and nine cases of tubercular disease (Galton, 1883). (b) Composite images of 22 individuals 2 hours after an injection of a placebo (left) or a bacterial endotoxin (right). Note the subtle change in expression after the toxin. Reproduced from Axelsson et al. (2018), *Proceedings of the Royal Society B: Biological Sciences* published under creative commons. Permissions for reproduction were obtained from https://www.copyright.com/. The author's permission was provided by email.

**Figure 2.**
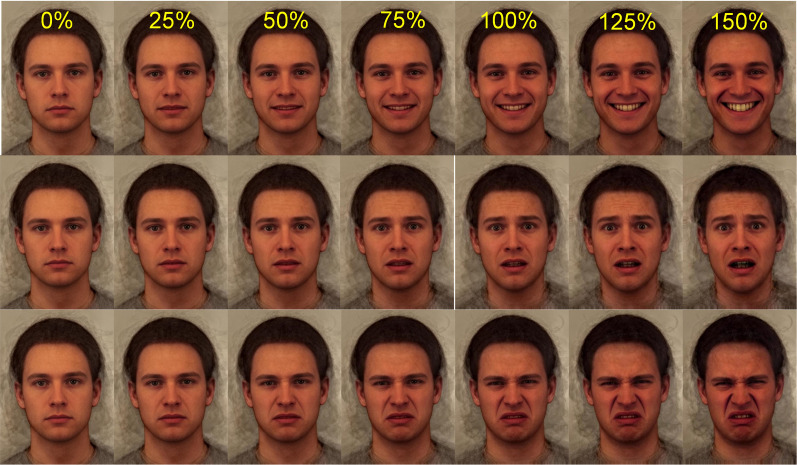
Composite faces posing happy, afraid and disgust expressions. Row 1: a composite of images of 35 males posing with neutral expression (0%) and a happy expression (100%). The differences in shape, colour and texture between the neutral and happy face images are used to transform the neutral image in 25% steps. This creates images where the happy expression gradually emerges in intensity. The 125 and 150% images represent an extrapolation of the series with caricature exaggeration of the happy expression by 25 and 50%. Row 2: as row 1 for the expression of fear. Row 3: as row 1 for the expression of disgust. Composite images and emotion transforms produced by the author.

**Figure 3.**
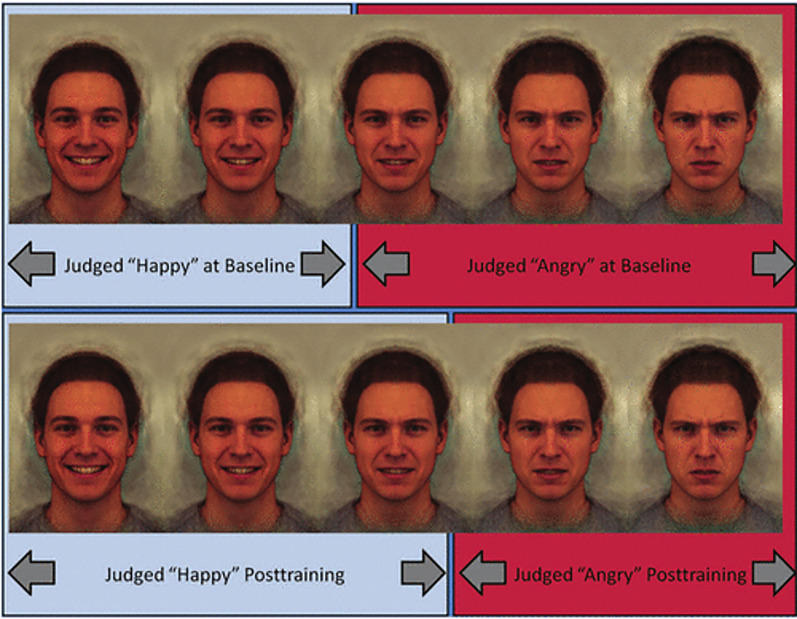
Happy to angry facial expression continuum. Five steps are illustrated progressing from 100% happy to 100% angry. The central image is ambiguous showing both characteristics of happiness and of anger. The upper part of the figure illustrates the categorical boundary between the images being categorised as angry or happy before training (see text). The lower section illustrates that, post training, the boundary is shifted such that more ambiguous expressions are classified as happy. Reproduced from figure 1 in Penton-Voak et al. (2013) *Psychological Science*, *24*, 688–697 with permission from the author.

**Figure 4.**
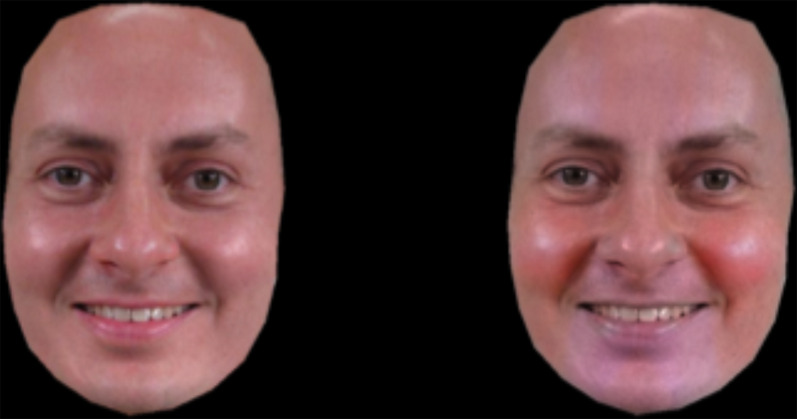
A face with and without additional diagnostic colour information for the emotion of happiness. With the augmented colour information, the images were easier to classify as happy. Reproduced under Creative Commons License cropping the original image to show only the face pair from figure S6 from Benitez-Quiroz et al. (2018) *Proceedings of the National Academy of Sciences*, *115*, 3581–3586. With permission from the author.

**Figure 5.**
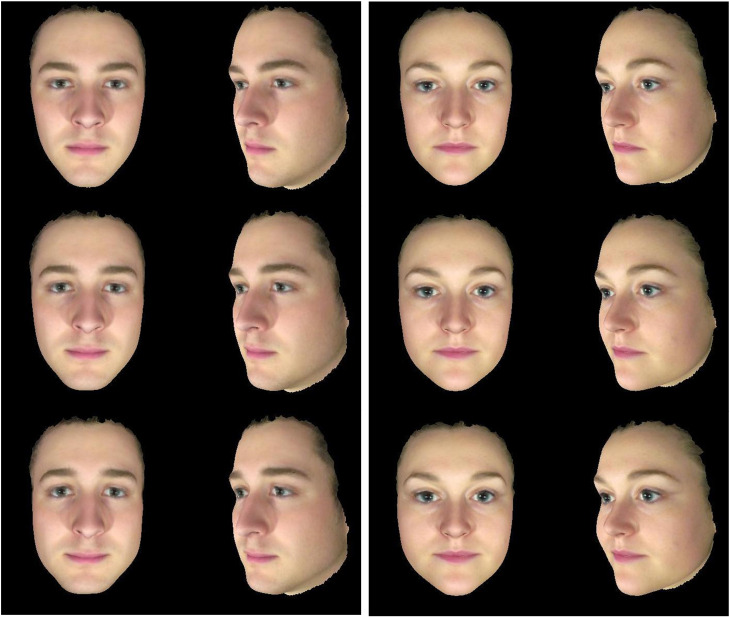
3D faces varying in apparent trustworthiness. Frontal and half profile views of male and female 3D head models varying in apparent personality. The head models were constructed by averaging together the 3D surface shape and texture of male and female faces separately (middle row). A collection of 118 faces (male = 50, female = 68) were rated for how trustworthy they looked while being rotated to reveal their 3D structure. For each gender, an average 3D head shape was formed from those faces that appeared high in trustworthiness. Separately an average was formed from those that appeared low in trustworthiness. These two averages defined a trustworthiness trajectory in 3D shape space for men and for women. Male and female composite faces were then transformed in shape along this trajectory to decrease apparent trustworthiness (top row) or to increase apparent trustworthiness (bottom row). Methods for averaging and transforming have been presented elsewhere (Holzleitner et al., 2014). 3D head models and apparent trait transforms models produced by the author.

**Figure 6.**
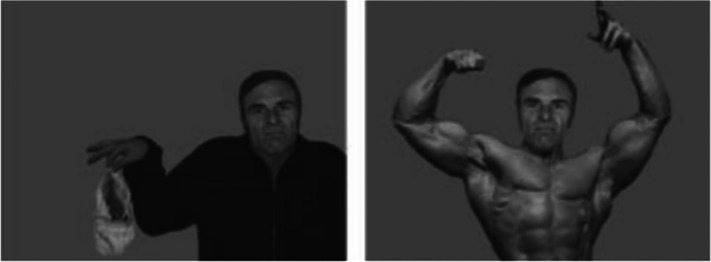
Disgust expression modified by context. An isolated facial expression of disgust was placed in a ‘disgust’ context (left) or in a ‘pride’ context (right). While the disgust expression was accurately categorised as negatively valanced in the disgust context, it was never categorised as having a negative valence in the pride context. Reproduced from figure 4a, Aviezer et al., (2008) *Psychological Science*, *19*, 724–732 with permission from the author. Permissions for reproduction were obtained from https://www.copyright.com/. The author's permission was provided by email.

**Figure 7.**
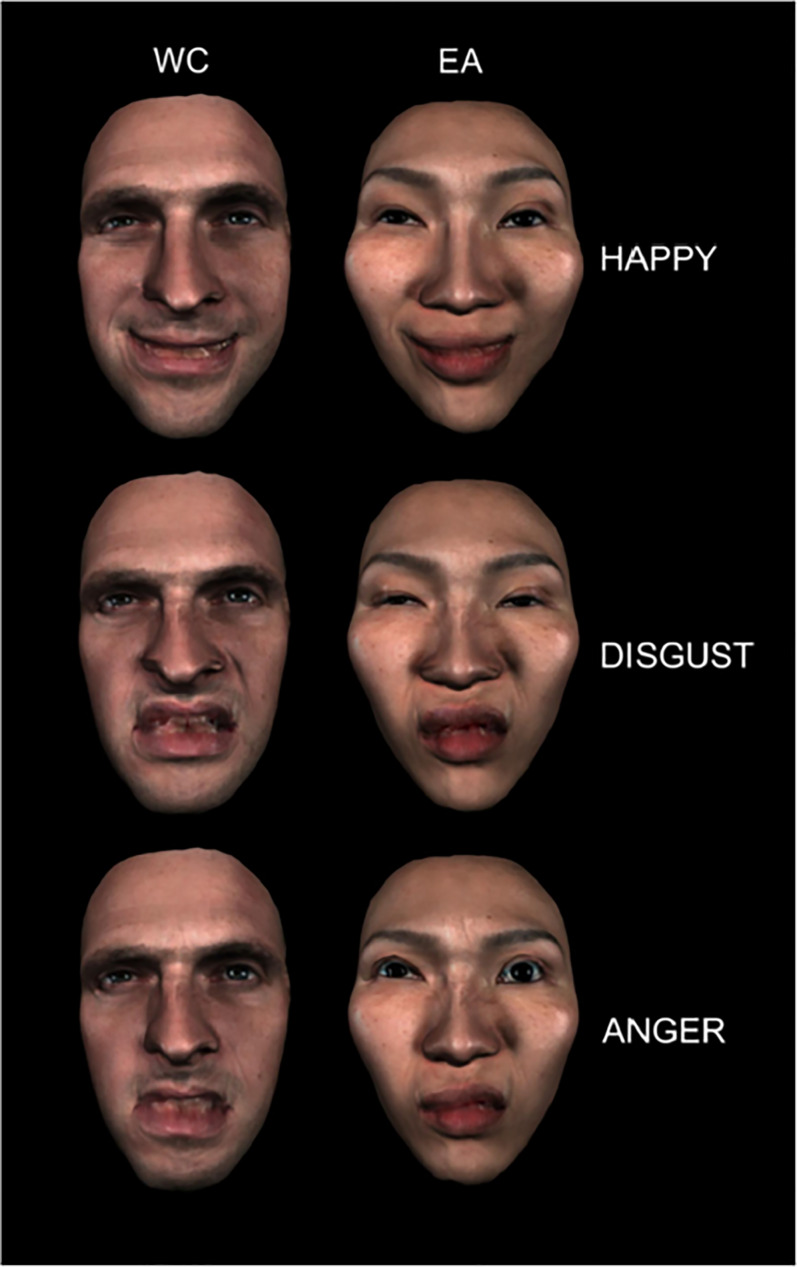
Comparing the representation of three expressions for one European (left) and one Chinese participant (right). The mouth region is more informative for the European and the eye region is more informative for the Chinese participant. Reproduced from Movie S2, Jack et al. (2012) *Proceedings of the National Academy of Sciences*, *109*, 7241–7244 with permission from the author. For the dynamic movie see http://www.pnas.org/lookup/suppl/doi:10.1073/pnas.1200155109/-/DCSupplemental/sm02.avi.

In Darwin's time, lithographs were the standard alternative to photographs (see Figures 1–21, Darwin, 1872), but photographic images offer greater verisimilitude. A lithographic engraving, however skilled, may exaggerate or fail to capture particular details that are present in real life or in the photograph on which they are based. Of course, photographs are not infallible; they too can miss particular expressive details because of perspective view or lighting shadows. Camera perspective can also modify the apparent expression of the face, e.g. when the head is bowed slightly, the chin appears smaller and less threatening (Zhang et al., 2020). Photographic capture may also be timed poorly relative to the apex of a dynamic expression. Yet despite each of these shortcomings, photography increases the accuracy of representation over lithographs.

## Multiple emotions

Darwin grouped emotional expressions into six categories. Ekman, a century later, concurred that a basic set of six emotions (happiness, surprise, fear, sadness, anger and disgust combined with contempt; Ekman et al., 2013) or seven (these six emotions plus interest; Ekman, 1972) are recognised universally. Others have argued that emotional expressions are not recognised with cultural universality (Russell, 1994). Some authors have argued that the number of expressions that are recognisable is likely to be more than the basic six (see Keltner et al., 2019). Others have argued that fewer than six are recognised across cultures (Jack et al., 2016). Perhaps part of the problem is that there is not 100% agreement in the labelling of facial expressions of emotions in any society. For example, using perhaps the most standard set of expression photographs from the Facial Expressions of Emotion Stimuli and Test, normal adult recognition accuracy of all 10 examples of each of six emotional expressions is about 80% (Young et al., 2002). Data for 227 individuals (aged 20–70 years with IQs of 90 and above) were used to define the average and spread of recognition performance. These data provide a cut-off score defining the border between normal range and impaired performance for each emotional expression (i.e. significantly different from the mean, *p* = 0.05; Young et al., 2002). For individual emotional expressions such as fear, a recognition score of 4/10 would lie at the boundary of normal and abnormal recognition. While 40% is higher than chance (1/6 or 16.7%), it is not particularly impressive. Indeed, the cut-off drops further to 3/10 (30% correct) when considering individuals older than 60 years. So, photographic examples of the most standard facial expressions are not that well recognised even within a single culture. Indeed, Barrett (2011) argues that the photographs of these prototypical expressions the actors have adopted (often muscle by muscle) are culturally agreed symbols of emotions (somewhat similar to emoticons 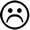) rather than being actual expressions that we come across in everyday use. The symbolic nature of posed expressions becomes more apparent from the work of Dawel et al. (2017). These investigators asked observers to judge posed expressions on a 15-point scale (–7 = completely fake; 0 = don't know; +7 = completely genuine). The majority of the posed expressions from standard expression sets such as those of Young et al. (2002) were not seen as genuine despite the fact that the same expressions could be correctly categorised as representing the emotional states angry, disgusted, fearful, sad and happy. The issue remains as to why expression recognition is not better but, as we will see, recognition of emotion depends on a variety of factors, including context.

## Composite portraits

Following on from Darwin's work on facial expressions, the technique of composite photography was developed in the nineteenth century. Composite construction is detailed here because it has had a pervasive influence on facial expression research. Darwin's cousin, Francis Galton (Galton, 1878, 1879), was a pioneer in making composite photographs. Galton sought to extract the generic features of different groups or types. His notorious views on eugenics may have been the drive to differentiate socially inferior types of man from healthy types (Levy & Peart, 2004). With the visual specification of healthy types one could then ‘encourage as far as practicable the breed of those who conform most nearly to the central type, and to restrain as far as may be the breed of those who deviate widely from it’ (Galton, 1907). Taking multiple exposures of different faces with each face aligned by the pupils of the eye would progressively average out idiosyncratic features and emphasise features common to faces in the group. For example, Galton combined photographs of convicted felons in an effort to generate the average criminal face. His drive here was consistent with his views on eugenics to breed out unwanted traits like criminality once he had identified what constituted a criminal type (Green, 1984), although had he succeeded in his endeavour, it would also be tempting to supply the resultant image to the police force who could then pre-emptively round up any would-be criminal on the basis of their facial appearance. By his own admission, the resulting composite did not look criminal, instead ‘the features of the composites are much better looking than those of the components. The special villainous irregularities in the latter have disappeared and the common humanity that underlies them has prevailed’ (Galton,1878). In short, the image appeared to be a man who looked quite handsome. Galton speculated that the composite might represent ‘the man who is liable to fall into crime’, which is hardly useful as this might apply to anyone given particular circumstances.

The finding that the process of averaging multiple faces increases the attractiveness of the resulting image has been upheld by modern Psychology (Langlois and Roggman, 1990). The evidence for this contention has improved with the development of computer graphic techniques (Little & Hancock, 2002). Practical tips on using such software to modify facial appearance are given by Sutherland et al. (Sutherland & Young, 2015; Sutherland et al., 2017). Computer composites are now made in several stages. First the structure of each component face image is defined with landmarks placed at corresponding feature points (e.g. the tip of the nose). The average face shape across the group is then calculated as the mean positions of the landmarks. Next, each component face image is reshaped to the average face shape. Finally, the colour and texture information from the corresponding positions in the reshaped images are averaged together (Tiddeman et al., 2001). The resulting composites are neatly in focus and appear lifelike, with no particular identity showing through (see Figure 1b).

The notion that beauty is, to a large extent, averageness might appear an enigma because it implies that beauty is nothing special. In fact, although the average-shaped face is indeed attractive, it is not *the* most attractive face shape (Perrett et al., 1994; DeBruine et al., 2007). Indeed, several factors that move faces away from averageness contribute to facial attractiveness; these include femininity, apparent health, youthfulness and positive expression (Perrett et al., 1998; Jones et al., 2004; Fink et al., 2006; Tatarunaite et al., 2005).

Galton also made composite images of healthy individuals (army personnel) and those suffering from disease (e.g. tuberculosis) (Galton, 1883; Figure 1a). His composites of sickness do suggest a pallor associated with consumption. With computer averaging, composite images can be produced with greater clarity, making it possible to read subtle expression changes accompanying sickness, as well as changes in pallor. Figure 1b compares the composite images of 22 volunteers 2 hours after the injection of either a placebo and/or a bacterial toxin. The reaction induced by the toxin includes a slightly drawn expression with downturned mouth and drooping eyelids, in addition to the skin losing redness and the lips becoming bluer (Axelsson et al., 2018).

## Computer graphic manipulations of expressions

A given expression has characteristic cues but these different cues may not be equally evident in all people making the expression. One way of depicting a typical emotional expression is, again, to generate a composite, averaging together many examples of that expression. Figure 2 illustrates the combination of 35 male faces from the Karolinska Directed Emotional Faces (KDEF) collection (Lundqvist et al., 1998). In Figure 2 top row, far left column, all of the men have a neutral expression whereas in the fifth column of the top row all of the men have a happy expression. While composite expressions are more representative than a single actor's pose, composites suffer from the same criticism raised by Barrett (2011). Each male actor displayed what they took to be the agreed symbol of the emotion whereas what is expressed in natural circumstances can differ.

Computer graphics can be used to enhance expressions in calculated steps. First the structure of a neutral or resting face is defined by a collection of (200) landmarks allocated manually to the corners of the mouth, the eyes and so on. The procedure is then repeated for a given expression, for example happiness (Figure 2 row 1, column 5). The difference between the position of the two corresponding sets of landmarks can then be measured. For example, with a smile present in a happy expression, one would expect the corners of the lips to be raised by contraction of the zygomatic muscles. Additionally, during a genuine smile the eyelids would close slightly owing to constriction of the orbicularis occuli muscles. Indeed, there would be a whole configuration of changes in the position of each of the facial landmarks. With computer graphics the landmarks are used to deform the photographic image, much as a sheet of rubber can be stretched by anchors at given points. The movement of the landmarks can be exaggerated (or diminished) computationally. The result is an expression of hyper happiness for the caricaturing exaggeration (125 or 150% Happy in Figure 2). Alternatively, diminution of the movement anchors results in a more subtle (25 or 50%) smile.

This technique has been used extensively in neuroscience in attempts to map out the brain systems involved in analysis and recognition of particular emotions. For example, the amygdala becomes more activated as expressions move between neutral to fear and exaggerated fear (Figure 2 row 2; Morris et al., 1996). Likewise, the insular cortex shows increasing blood flow as expressions viewed move from neutral through disgust to exaggerated disgust (see Figure 2 row 3; Phillips et al., 1997). Thus, computer exaggeration of facial expressions has enabled various brain systems to be implicated in the processing of emotions.

The use of standardised expressions of emotion has also revealed problems in emotion recognition. Such testing can involve the presentation of full-blown expressions, yet some emotions such as happiness are relatively easy to recognise and performance is therefore often at the ceiling (i.e. 100% correct). A more sensitive way of testing recognition has been to employ subtle expressions produced by computer graphic transformation of neutral faces in small steps towards the full expression (e.g. 25, 50 and 75% expressions in Figure 2, columns 2–5).

The misperception or failure to recognise individual or multiple expressions of emotions, particularly at low intensity levels, has been associated with a variety of conditions following brain injury or neurodegenerative diseases, including amygdala damage (Adolphs et al., 1999), Huntington's disease (Sprengelmeyer et al., 1996), Wilson's disease (Wang et al., 2003) and Parkinson's disease (Coundouris et al., 2019). Recognition of emotion in facial expressions is compromised by a great range of issues affecting mental health, including autism (Law-Smith et al., 2010; Eack et al., 2015, although see Cook et al., 2013), alcoholism (Frigerio et al., 2002; Kornreich et al., 2002), alexithymia (Cook et al., 2013), bipolar disorder (Venn et al., 2004), borderline personality disorder (Daros et al., 2013), depression (Krause et al., 2021), psychopathy (Montagne et al., 2005; Hastings et al., 2008; Dawel et al., 2012), schizophrenia (Kohler et al., 2010) and social phobia (Bell et al., 2011). This list is extensive but is by no means exhaustive. It indicates the pervasiveness of problems in understanding expressions of emotion. Indeed, emotion recognition failures may exacerbate mental health problems by frustrating normal social interaction.

## Bias in interpreting ambiguous expressions

Another way that computer graphics have been harnessed to investigate the perception of emotions is to ‘morph’ or dissolve slowly from one expression to a different expression (e.g. in Figure 3 a happy expression is changed in stages to an angry expression). Despite the continuous nature of the morphing process, the way we interpret the images is discontinuous or categorical. We are likely to label all the images on the left of the sequence as belonging to the category ‘happy’ and the steps on the right of the sequence as belonging to the category ‘angry’. At some point along the continuum the category switches. Images at this point are the most ambiguous and have the longest reaction times for observers to assign them a name or category (Young et al., 1997).

The ambiguity of the facial configuration at the midpoint or categorical boundary of two expressions is itself valuable as it can be used to demonstrate biases in the identification of emotions. In depression there is a bias to interpret ambiguous emotions negatively. Depressed individuals are more likely to report ambiguous or mixed expressions as sad and less likely to report them as happy (Bourke et al., 2010).

For juvenile offenders with conduct disorder there is often a bias to see others as threatening (Dodge et al., 1990). This hostile attribution bias is rational in an adverse environment but may be self-reinforcing. In a vicious cycle, a negative interpretation of another's expression can lead to an aggressive reaction which is in turn reciprocated. Penton-Voak et al. (2013) reasoned that it may be possible to reverse the process by establishing a virtuous cycle where a shift in the interpretation may allow positive reactions to be reinforced.

Penton-Voak et al. (2013) used intermediate or ambiguous expressions in an attempt to rehabilitate conduct disorder. Adolescent participants were given a task to decide whether an image was happy or angry. The stimuli presented were a series of images gradually morphing between anger and happiness (see Figure 3). The participants received biased feedback. When they identified ambiguous stimuli as happy they received ‘correct’ as feedback, reciprocally when they labelled the ambiguous emotion as angry they received ‘incorrect’ as feedback. After training, the category boundary between angry and happy expressions shifted so that more intermediate expressions were classified as happy. Of greater significance, participants reported less anger and staff reported less aggressive behaviour in the two weeks after training (Penton-Voak et al., 2013). The malleability of emotion categories used to label the same facial expression reinforces the role of learning in the interpretation of emotions (Barrett et al., 2019).

## The colour of happiness

Benitez-Quiroz et al. (2018) claim that colour can be used to support recognition of facial expressions of emotion. This capacity to use colour for emotion recognition may depend on guessing the valence of the underlying emotion with happiness representing positive valence and anger, fear, disgust and sadness all representing negative valence emotions. The strongest colour cue comes from happy expressions because of the blood flow and reflection changes in the cheek area. Hence, red cheek colour is a cue to a positively valanced emotion. The diagnostic value of colour shows the importance of colour photography in the reproduction of facial expressions. Of course colour use adds to the expense of printing.

## Adding depth to emotions

A development in representation of faces with emotional expressions comes from the shift from two to three dimensions. A three-dimensional model also allows measures of responses to facial expressions aimed towards or away from the observer. One can determine the cone of view in which people judge expressions to be directed at them. Most observers have an egotistical bias and interpret someone's happy expression as directed at themselves while more negative emotions are interpreted as directed away from them and towards someone else (Lobmaier & Perrett, 2011). The tendency to see negative expressions as self-directed is heightened in people who are socially anxious and could exacerbate their anxiety (Schulze et al., 2013).

To view items in 3D, it is common to think of stereoscopic displays where the left and the right eyes receive separate views of two different photographs. Publications with such requirements are clumsy (e.g. issuing red–green viewers and printing two photographs in red and green ink; Julesz, 1971). Alternative strategies for displaying 3D images include the use of motion parallax, for example, by presenting a video of the head rotating (Holzleitner & Perrett, 2016).

The advantage of a 3D head model is that it allows viewing from more than one perspective. This advantage can be achieved by rendering two or more 2D views of the 3D model and presenting them together. The aim here is not to achieve a stereoscopic view but to provide readers with the increased visual information that comes from the 3D model. Viewers can be presented with the front view and also a view turned towards the profile. The combined views give more information about the chin, nose, brow and forehead than is available from a frontal 2D image Figure 4 (see Figure 5 for example).

## First impressions

We readily make judgments of a person's character even when we have not observed any behaviour. While there are many adjectives that we might use to describe a person's character (intelligent, dominant, warm, charismatic and so on), these judgments can be boiled down to just two or three dimensions (Todorov et al., 2008; Sutherland et al., 2013). The most important of these dimensions reflects emotional expression. The first dimension of character judgment is referred to as trustworthiness, warmth and sometimes valence. A person with a slightly positively valanced emotional expression (with upturned lip corners and slightly raised eyebrows, see Figure 5 bottom row) is seen as trustworthy (Todorov, 2008). Reciprocally, a slightly negatively valanced emotional expression (i.e. with downturned mouth and lowered eyebrows) is seen as untrustworthy (see Figure 5 top row). This trustworthy dimension refers to a person's intentions: warmth and approachable vs. hostile and unapproachable.

A second dimension is often referred to as power or competence and may reflect the impression of capacity to carry out intensions. This dimension is most strongly linked to maturity and masculinity. A large male with masculine physique has the power to act antagonistically and with impunity, whereas a baby-faced adult looks weaker in physique and seems to have little power to act dangerously even if angry.

Face images are often collected with individuals posing with a neutral expression (e.g. Todorov et al., 2008), nonetheless the apparent expression of neutral or resting faces varies considerably. A person's mouth may appear to curve down or upwards depending on underlying anatomy or posture. Likewise, eyebrows may appear lowered or raised, depending on anatomy and pose. These subtle differences in apparent expression play a major role in the attribution of personality (Todorov, 2008). Zebrowitz et al. (2010) found that individuals who displayed a resting expression resembling anger were seen as less trustworthy and less competent. Resemblance to expressions influenced trait impressions even when statistically controlling possible confounding influences of attractiveness and baby-facedness.

It is important to note that trait attributions need not show any relation to actual personality. Furthermore, we have systematic biases when interpreting expressions and other facial cues. As noted, we assume that angry-looking individuals are less trustworthy even though there is no causal link between trustworthiness and the tendency for anger. Indeed, there is a danger that artificial intelligence software for classifying facial expressions of emotion (Pantic & Rothkrantz, 2000) will be misused to differentiate presumed trustworthy and untrustworthy individuals. Attempts to classify people into different types from face photographs is a misguided endeavour and will unwittingly reinforce the biases that we hold (see y Arcas et al., 2017; Bowyer et al., 2020). For example, individuals convicted of crime may be more likely to come from lower socio-economic sections of society. Any cue related to low socio-economic status, e.g. low health or mood at the time of photograph, could contribute to criminal face image classification even when the cue is not causally linked to crime. The sources of images of criminals are likely to be systematically different to sources of images for non-criminals. Comparing mugshots of supposed criminals taken at a police station with web-based images of supposed non-criminals would be an obvious error but even when this difference in photography is controlled for there are further confounds (Bowyer et al., 2020).

## Emotion in context

Aviezer et al. (2008) placed isolated facial expressions of disgust on the body of an actor/actress in different contexts (displaying disgust, anger, fear and sadness). Participants were greatly affected by the context and frequently miscategorised the emotion. For example, in the angry context participants labelled the disgust facial expression as anger on 80% of trials. In a second experiment, disgust face expressions were placed in a negatively valanced disgust context (e.g. a man holding soiled underwear) or in a positively valanced pride context (e.g. a body builder showing off his muscular torso, see Figure 6). Eighty per cent of disgust expressions were categorised as negatively valanced in the disgust context but 0% of the disgust expressions were categorised as having a negative valence in the ‘pride’ context (Aviezer et al., 2008). This experiment makes it clear that facial expressions of emotion are not interpreted in isolation from other environmental cues, including body posture. Meeren et al. (2005) note that ‘When face and body convey conflicting emotional information, judgment of facial expression is hampered and becomes biased towards the emotion expressed by the body’. Indeed, the accompanying environmental context presented alongside a facial expression is remembered better when observers are able to categorise the emotion displayed (Barrett & Kensinger, 2010). That is, context is key to understanding and remembering the displayed emotion (Barrett et al., 2011).

Thus, one aspect of ecological validity in presenting expressions of emotions is to provide an environmental context, and to include information from body posture. Perhaps that is why Darwin chose to display both the face and the body in many his photographic plate illustrations of emotions (Darwin, 1872).

## Dynamic expressions across cultures

Darwin (1872) sent a questionnaire to variety of colleagues abroad to examine the extent of commonality of expressions across different societies. He argued for consistency in expressions and their recognition across cultures and, indeed, reviews of cross-cultural perception have concluded that there is considerable commonality across peoples (Elfenbein & Ambady, 2002; Sauter & Eisner, 2013). More recent assessment across cultures suggests that there is also diversity in interpretation (Gendron et al., 2018). Evidence for some diversity in facial expressions has come in part from research using dynamic expressions (Jack et al., 2012). Jack et al. (2012) showed 15 European and 15 Chinese participants’ dynamic facial images with combinations of different muscles contracting to build up a visual representation of the expression of each of the six basic emotions. In general, there was more information about the nature of the expression from the mouth and lower parts of the face for the Western participants and conversely more information about the expression from the eyes and upper part of the face for the Eastern participants (see Figure 7). For the European observers, the six expressions appeared to be based on unique sets of muscle contractions. For Chinese observers, the muscles used for the different expressions were less consistent and showed some overlap across the six emotions. For happy, angry and disgust expressions Chinese participants represented the intensity of emotion with movements of the eye region while this was less true for the European participants. Hence Jack et al. (2012) concluded that expressions vary with culture.

Regardless of the commonality or diversity in expression interpretation, Jack's research shows how the methods of expression illustration have developed. The expression stimuli used by Jack et al. (2012) included internal facial movement. The skin and features were made to deform in a manner corresponding to those that take place when a given face muscle contracts, holds and relaxes. Chen et al. (2018) illustrate similar methods for combining different facial muscles contracting with different time courses (http://movie-usa.glencoesoftware.com/video/10.1073/pnas.1807862115/video-1). Chen et al. (2018) also show the process of averaging together different instances of the same class of dynamic expression (https://movie-usa.glencoesoftware.com/video/10.1073/pnas.1807862115/video-2). With these methods Chen et al. (2018) show cultural generality in the mental representations of pain and culturally different ‘accents’ in the representations of pleasure (orgasm).

## Future illustrations

Darwin's book, *The expression of the emotions in Man and animals* (1872), was one of the first mass-market books to contain photographs. The publisher John Murray warned Darwin that including the photographs would ‘would poke a terrible hole in the profits’ of the book. Costs have always been a factor to consider when printing illustrations. Over the last few decades, authors have often had to bear the cost of colour printing and reproduction. Two to four colour illustrations could have easily run into thousands of pounds, which has been prohibitive for many. With the rise of online publishing, dynamic and colour images should be more accessible, as there should be no extra cost for reproducing these online.

More specifically, there is no reason why publications cannot include 3D images that have depth through motion (e.g. rigid rotation about one or two axes) or through dynamic internal changes to shape (e.g. Holzleitner & Perrett, 2016, extras, https://ars.els-cdn.com/content/image/1-s2.0-S1090513815001208-mmc5.mp4). These films (mp4 clips) are currently included in appendices or links but when publishing electronic versions there is no reason not to include the movies within the text – something like the *Daily Prophet* in the Harry Potter films. While technological development might be needed to allow holographic representation, portrayal of depth can easily be achieved with current technology. Some journals have begun to publish video presentations of methods, for example the *Journal of Visual Experiments* (https://www.jove.com/), but no journals are integrating videos into text in a manner that can be found in newsfeeds.

All of the expression images described here have been based on images captured from real faces. Computer graphic techniques have made huge progress in building models of faces and synthesising expressions on the models. In many cases an actor may drive the 3D representation as an avatar to produce the correct expression dynamics. To achieve this, landmarks are placed on an actor's face around the eyebrows and mouth, etc. The landmarks are filmed at high speed and resolution in two or three dimensions. The movement trajectory of each landmark can then be used to drive the corresponding landmark on a 2D or 3D model of a face (Tiddeman & Perrett, 2002; Cao et al., 2013). Automated facial feature finding allows the same to be achieved for actors without them wearing landmarks, with only a slight loss of fidelity. The avatar model can be a different identity, sex, age or species as long as the corresponding landmarks can be found. The avatar can also be a transformed version of the actor, for example the actor depicted with increased age. In effect, this process systematically transfers facial movements and expressions onto the avatar. The avatar retains some of the actor's identity because idiosyncrasies of expression are transposed from the actor to the avatar.

The models of faces driven are increasingly realistic and include skin properties such as albedo, elasticity, dynamic texture and sub-surface light scattering (Weyrich et al., 2006; Chandran et al., 2020; Chen et al., 2020). Facial representations have passed through the uncanny valley where being approximately correct looks weird (Mori et al., 2012); we are now in a state in which we can no longer differentiate reality and (re)construction (e.g. https://www.youtube.com/watch?v=HjHiC0mt4Ts). Typing hyper-realistic facial animations into a search engine will show the latest developments (e.g. https://www.youtube.com/watch?v=W_rphISMMzs&list=TLPQMTQxMTIwMjEqrW9Bb9oJAQ&index=3). With realism comes believability and a new problem of ‘deepfakes’. It is relatively straightforward to create an avatar that has a likeness to anyone, be they a celebrity or national leader. Convincing deepfakes are built from a corpus of utterances and expressions made by the target (see https://www.youtube.com/watch?v=p1b5aiTrGzY). The impersonating avatar or deepfake can then be controlled to say or do just about anything, complete with idiosyncratic facial expressions and mannerisms (e.g. https://www.youtube.com/watch?v=ttGUiwfTYvg). Techniques for detecting and mitigating the effects of deepfakes are under development but deepfake videos are already of sufficient quality to attract millions of viewers, many of whom accept their authenticity (Kietzmann et al., 2020). Such realism has yet to make it through to research on facial expression. When it does, we can expect to see ever greater contextual effects and individual differences in the nuances of interpretating emotions.

One of the characteristics of individuals with schizophrenia (e.g. Jeannerod, 2003) is that they are less able to differentiate movements or voices created through their own actions from those created by others (Jeannerod, 2003; Johns et al., 2001). In effect, individuals with schizophrenia are less able to discriminate self from others. While these are clinical symptoms there are parallel problems that could potentially be introduced by technology.

It will not be long before avatars become used as our representatives in work and in social interactions. As explained, technology allows our dynamic facial expressions to be mimicked on an avatar. It is not a big step for the avatar's emotional expression to be caricatured so that a look of mild displeasure may become amplified to outright anger. Conversely, the same expression could be muted so that the avatar's repertoire allows full anger management in the face. Further, one can imagine tweaking the baseline emotional disposition from normal to a cheerier or a more sullen avatar. Such control could allow individuals to be more assertive, or more friendly, than they are in real life.

These advances seem potentially beneficial, yet there are all too obvious dangers. With a new wave of avatars acting and expressing on our behalf, there are likely to be inaccuracies and biases in the sense of self and sense of ideal. Indeed, there is plenty of evidence that, in the domain of body shape, the divergence of people's sense of self and their sense of ideal has been to the detriment of body image, and has encouraged eating disorders, excessive exercise and steroid use (Derenne & Beresin, 2006; Harvey & Robinson, 2003). The move to expressive avatars representing us in social interactions is likely to bring with it a new set of problems.

## Future directions

There has been a plethora of research on the perception of static expression photographs. Work with dynamic expressions is developing (Dawel et al., 2021), but there are many more avenues to explore with dynamic expression stimuli. For example, how do the dynamics of expression affect attribution of personality?

It is important that assessments of expression recognition in clinical groups be made with dynamic facial expressions that are perceived as genuine. Spontaneous genuine (dynamic) expressions are easier to discriminate compared with artificially posed static expressions (Namba et al., 2018). Most of the facial expression stimuli that have been used to date are not perceived as genuine. Thus, our current understanding of how mood, personality and clinical conditions affect the interpretation of emotional expressions in social interactions is sure to need revision.

Dawel et al. (2021) make a plea for studing natural human expressions made ‘in the wild’ rather than studying synthetic expressions made by avatars. There is a need for study of both since, like it or not, we are going to be experiencing many more synthetic faces in the near future. While it is likely that the use of synthetic expressions could be detrimental, there are also avenues of potential benefit.

Questions such as how many expressions are recognised and what factors (e.g, culture) account for variance in recognition have already been addressed. With new technologies, it is important to address new questions, particularly those concerning the experience of seeing oneself and others making emotional expressions on avatars. Do we feel a different intensity of positive expression when portrayed by a predominantly happy or grumpy avatar? Can the prevailing mood of an avatar we control influence our own mood? If so, this would suggest avenues to explore for therapy of mood disorders. Can avatar expressions be exaggerated or made more typical in real time to benefit individuals with interpretation difficulties (e.g. alexithymia or macular degeneration, Lane et al., 2019)?

A welcome recent trend has been for journals to require that data and stimuli be made freely available from repositories. Expression databases and facial control programs (e.g. KDEF, Lundqvist et al., 1998; The Emotion Recognition Task, Montagne et al., 2007; FEEST, Young et al., 2002; FaceGen, Todorov et al., 2008; Psychomorph, Tiddeman et al., 2001; Sutherland et al., 2017) have helped research by providing both standardisation and flexibility in use by non-computer scientists. The community needs equivalent openness in access to expressive avatars that can be driven easily and equivalently by different research groups. Such facilities are likely to emerge to service appetites for their use in social media.

## Research transparency and reproducibility

A data availability statement: there are no data accompanying the manuscript. Illustrations have been prepared with Morphanalyser 2.4 https://cherry.dcs.aber.ac.uk/morphanalyser/version2.4/launch2.4.html and Java Psychomorph https://users.aber.ac.uk/bpt/jpsychomorph/
